# 2521: Research participant 101: What you need to know before joining a research study

**DOI:** 10.1017/cts.2017.184

**Published:** 2018-05-10

**Authors:** Victoria Straughn, Erin Haynes, Emma Jones, Jacqueline Knapke

**Affiliations:** University of Cincinnati, Cincinnati, OH, USA

## Abstract

OBJECTIVES/SPECIFIC AIMS: The goal of this innovative course is to provide community members with sufficient information to either join or decline participation in clinical research. We anticipate that they will gain knowledge in why research is conducted, the ways participants are recruited, the history of research, regulations that guide research today, participant protections, understand the consent process, their risks and benefits of participating in clinical research. METHODS/STUDY POPULATION: We will recruit interested community members via flyers placed at the training location and at other local community centers and agencies that receive heavy foot traffic. The course is listed in the Communiversity catalogue which is distributed in hardcopy (over 30,000) and email each semester. The course will be taught by a longstanding community member and research coordinator at the University of Cincinnati. Each session will be highly interactive including videos, role-play, and discussion of the presented research topics. Evaluation will occur both pre and post-session, along with pre and post-course. RESULTS/ANTICIPATED RESULTS: We anticipate 20–30 participants at each of the 4 sessions. We anticipate that we will learn current perceptions of clinical research and barriers to their participation to enable improved research recruitment. In addition, we will gain new insights into clinical research needs of the community. DISCUSSION/SIGNIFICANCE OF IMPACT: Through these interactive sessions, we will learn why community members participate in research and their barriers to participating. Understanding the perception of research by the target community is critical when developing clinical research recruitment strategies. We will also be developing a more educated community towards clinical research. We will also gain great insight into new clinical research directions as indicated by community members.

